# Characterization of a PNLIP variant identified in Amish pediatric patients with congenital pancreatic lipase deficiency

**DOI:** 10.1016/j.jlr.2025.100878

**Published:** 2025-08-19

**Authors:** Grace E. Curry, Nicole L. Bertsch, Tran Quach, Rhonda Anderson, Neel Matiwala, Karlla W. Brigatti, Steven J. Wilhelm, Katie B. Williams, Mark E. Lowe, Zineb Ammous, Xunjun K. Xiao

**Affiliations:** 1 Department of Pediatrics, Washington University School of Medicine, St. Louis, MO, USA; 2 The Community Health Clinic, Shipshewana, IN, USA; 3 Clinic for Special Children, Gordonville, PA, USA; 4 Center for Special Children at La Farge Medical Clinic, Vernon Memorial Healthcare, La Farge, WI, USA

**Keywords:** Dietary fat, Digestive lipase, fat maldigestion, fat-soluble vitamin deficiency, nutrition, pancreatic lipase, protein misfolding, steatorrhea, vitamin A, vitamin D

## Abstract

Congenital pancreatic lipase deficiency (CPLD, OMIM #614338) is a rare exocrine pancreatic disorder presenting in late infancy with steatorrhea, fat-soluble vitamin deficiency, and low pancreatic lipase activity. Variants of the pancreatic triglyceride lipase (PNLIP) gene have been linked to CPLD. Six children from four Amish families exhibited CPLD symptoms, and two had decreased fecal elastase levels when tested. A novel homozygous *PNLIP* variant, c.869G>A (p.S290N), was identified in these children. This study aimed to characterize the PNLIP variant to understand its mechanism underlying CPLD. The variant impact was first evaluated using computational modeling. Functional analyses included activity assays, cellular PNLIP partition assessments, and endoplasmic reticulum (ER) stress evaluation in transfected cells. Computational modeling showed that p.Ser290 is highly conserved across species and the variant causes steric hindrance, resulting in protein misfolding. Functional assays revealed that the PNLIP variant had a complete loss of activity compared to the wild type (WT), with defects in catalytic function and secretion. Immunoblotting showed reduced PNLIP variant in the medium and increased accumulation in the detergent-insoluble fraction, consistent with protein misfolding. Variant-expressing cells had elevated levels of BiP, an ER stress marker, and increased *Xbp1* mRNA splicing, suggesting an elevated ER stress and unfolded protein response (UPR). In conclusion, the *PNLIP* p.S290N variant causes CPLD through a loss-of-function mechanism, characterized by loss of enzymatic activity and defective secretion due to protein misfolding. Further studies are needed to determine whether the misfolding variant protein induces proteotoxicity, potentially increasing the risk of pancreatic injury, including chronic pancreatitis.

Congenital pancreatic lipase deficiency (CPLD, OMIM #614338) is a rare medical condition typically presenting with symptoms such as steatorrhea, fat-soluble vitamin deficiency, and low pancreatic lipase activity beginning in late infancy (1, 2, 3, 4, 5, 6). It was only about a decade ago that molecular evidence first linked CPLD with variants in the gene encoding pancreatic triglyceride lipase (PNLIP) (7, 8, 9). The *PNLIP* gene is located on chromosome 10q26.1, and it comprises 13 exons and spans more than 20kb. The *PNLIP* gene encodes a 465-amino acid protein precursor that includes a 16-amino acid secretory signal peptide. After the cleavage of the signal peptide, the mature protein consists of 449 amino acids and has a size of approximately 50 kDa. PNLIP (EC 3.1.1.3) is one of the most abundant digestive enzymes secreted by the pancreas into the duodenum, where predigested dietary fats are mixed with bile salts and pancreatic juice (10, 11). Under normal physiological conditions, PNLIP digests fats efficiently with the assistance of its cofactor, colipase. PNLIP has been recognized as the main lipase for dietary fat digestion in adults and older children, primarily hydrolyzing triglycerides, the predominant dietary fats (12, 13). Two other pancreatic lipases, pancreatic lipase-related protein 2 (PNLIPRP2) and carboxy ester lipase (CEL), play some compensatory roles in dietary fat digestion (14). In newborns and young infants, PNLIP is either absent or expressed at minimal levels (15). During this period, CEL from mother's milk may compensate for the low PNLIP levels and work together with PNLIPRP2 and CEL produced by the infant's own pancreas to digest fats (16, 17). Although in rodents PNLIP starts to be expressed in the pancreas around the time of weaning, the precise timing of when PNLIP begins to be expressed in humans remains unclear (18, 19).

We have previously characterized several *PNLIP* variants associated with CPLD identified by others (20, 21). The variants result in a loss of function, with all variants exhibiting defective secretion (p.T221M, p.W419∗, and p.R188C) except one variant causing poor expression due to nonsense-mediated mRNA decay (p.W102∗). These pathogenic variants occurred in the homozygous or compound heterozygous state to cause clinical symptoms. Heterozygous carriers typically exhibit no clinical symptoms, suggesting an autosomal recessive inheritance pattern. Thus far, both cases of CPLD associated with homozygous *PNLIP* variants were from consanguineous parents found to be heterozygous carriers (p.T221M (7) and p.W419∗ (9)).

In this study, we characterized the structural and functional effects of a novel *PNLIP* variant, c.869G>A (p.S290N), identified in six children from four Old Order Amish families. We expressed PNLIP p.S290N in transfected cells to elucidate the mechanism for CPLD. The results show that the missense variant results in loss of lipase activity through protein misfolding in vitro. The misfolded protein triggers increased endoplasmic reticulum (ER) stress and activation of unfolded protein response (UPR), suggesting potential proteotoxicity. This finding has significant implications for the clinical care of affected patients.

## Materials and methods

### Nomenclature

For the human *PNLIP* gene, which encodes pancreatic triglyceride lipase, nucleotide numbering begins with +1 corresponding to the A of the ATG translation initiation codon of the coding DNA sequence. Amino acid residues are numbered starting with the initiator methionine of the primary translation product.

### Affected pediatric patients, their families, and clinics

This study included six affected patients from four Old Order Amish families. Five of the affected children (Patient # 1 and 2 from Family 1, Patient # 3 and 4 from Family 2, and Patient # 6 from Family 4) from Northeast Indiana were evaluated at The Community Health Clinic in Shipshewana, Indiana, due to stool abnormalities. Northeast Indiana, home to the third-largest Amish population in the US, has a high prevalence of genetic disorders in the local Old Order Amish community due to the founder effect (22). The other affected child (Patient # 5 from Family 3), from a family residing in Wisconsin but originally from Northeast Indiana, was evaluated for stool abnormalities at the Center for Special Children at the La Farge Medical Clinic of Vernon Memorial Healthcare, Wisconsin.

The Community Health Clinic, Center for Special Children, and Clinic for Special Children in Gordonville, Pennsylvania, are among a small number of nonprofit medical clinics that offer low-cost molecular genetic evaluation and medical care tailored to the needs of families from local Amish and Mennonite families with rare genetic and metabolic disorders. These clinics often collaborate with other institutions via research partnerships to provide cost-prohibitive clinical genetic testing, such as whole-exome sequencing. Parents provided written consent on behalf of their minor children for genetic testing according to Lancaster General Health protocol 095-2008. This study was approved by the Institutional Review Board at Washington University (IRB ID #: 202409015) and abided by the Declaration of Helsinki principles, and written informed consent was obtained from all participants or their parents.

### Whole-exome sequencing and targeted sequencing

Peripheral blood samples were collected from the affected patients and their family members, and genomic DNA was extracted from EDTA anti-coagulated blood samples using standard methods. Whole exome sequencing for the first set of affected siblings was performed through a research collaboration with the Regeneron Genetics Center and the Clinic for Special Children, as previously described (23, 24, 25, 26). Briefly, 1 μg of high-quality genomic DNA was used for exome capture, and the libraries were sequenced on the Illumina HiSeq 2500 platform using v4 chemistry, achieving coverage of more than 85% of bases at 20x or greater. Raw sequence reads were mapped and aligned to the GRCh37/hg19 human genome reference assembly using BWA/GATK bioinformatics algorithms. Called variants were assessed using standard metrics and subsequently filtered by observed minor allele frequency (≤1%) within public databases, Regeneron Genetics Center internal databases, and Clinic for Special Children population-specific allele frequency databases. The annotation of potential functional effects was based on in silico predictions and conservation scores from multispecies alignment. Classification of pathogenicity for candidate exome variants was based on the American College of Medical Genetics and Genomics guidelines. Whole-exome sequencing of the affected siblings identified a novel homozygous c.869G>A (p.S290N) variant in the *PNLIP* gene, using the reference sequence for *PNLIP* (GenBank: NM_000936).

The variant was further confirmed through targeted Sanger dideoxynucleotide sequencing. Briefly, the DNA segment flanking the c.869G>A variant site was amplified by polymerase chain reaction (PCR) using specific oligonucleotide primers: Forward: 5′-GACCTGCATGAGCTCACACTAA-3′; Reverse: 5′-GAAAGCACACCAGTCATGAGAA-3′. The resultant PCR product of 302-bp amplicon was then purified and sequenced using dideoxynucleotide sequencing to confirm the presence of the c.869G>A variant. The targeted sequencing approach was then used to genetically test the other affected children and their families.

### Computational modeling

The programs PredictSNP1, SAAPdap/SAAPpred, and AlphaMissense were used to test the structural impact of p.S290N (27, 28, 29). PredictSNP1 combines six prediction tools into a “consensus classifier for prediction of disease-related variants”. SAAPdap/SAAPpred employs a computational approach to determine whether a variant will be pathogenic. AlphaMissense is a machine learning approach that utilizes structure predictions by AlphaFold2 and population frequency data to predict the pathogenicity of variants on proteins. The PNLIP p.S290N structure was modeled using PyMOL Molecular Graphic System (Version 3.10; Schrodinger, LLC).

### Construction of plasmid DNA containing *PNLIP* S290N

The plasmid DNAs pcDNA3/*PNLIP* wild type (WT) and pcDNA3/*PNLIP* S290N were constructed following a previously described protocol (20). The variant vector, pcDNA3/*PNLIP* S290N, was generated by site-directed mutagenesis, using the pcDNA3/*PNLIP* WT plasmid as a template. The accuracy of the sequences was confirmed through dideoxynucleotide sequencing.

### Maintenance and transfection of human embryonic kidney (HEK) 293T cells

Culture maintenance and transient transfection of HEK 293T cells (Cat.: CRL-3216, ATCC) were carried out using a previously described protocol (30, 31). Forty-eight hours post-transfection, conditioned media were collected and replaced with 1.2 ml Opti-MEM I Reduced Serum Medium (Cat.: 31,985,070, Thermo Fisher).

### Sample harvesting and processing

After an additional 20 h in the Reduced Serum Medium (for a total of 68 h post-transfection), both culture media and cells were harvested as previously described (30, 31). The harvested cell pellets were then lysed with a 500 μl solution of RIPA buffer (Cat.: R0278, Sigma) supplemented with EDTA-free complete protease inhibitor (Cat.: 4693132001, Roche). Next, the cell lysates were fractionated into whole-cell lysates and detergent-soluble and -insoluble fractions as previously described (30).

### Lipase activity assay

The activity of lipase in the conditioned media or detergent-soluble fractions was measured using a 5-min pH-stat method as previously described (32). For each assay, 200 μl of conditioned medium or cell lysate sample was added into a 15 ml lipase assay emulsion consisting of substrate including 114 mM tributyrin (Cat.: T8626, Sigma), 27 mM trioctanoin (Cat.: T9126, Sigma), or 6.9 mM triolein (Cat.: T7140, Sigma), 4 mM sodium taurodeoxycholate (Cat.: T0875, Sigma), and an excess of purified recombinant human colipase (2 mg, 13 nM) (33). The lipase activity was expressed as U/ml, with one unit equivalent to one μmol of fatty acids released per minute.

### Protein immunoblot

Samples of the conditioned media, whole cell lysate, and detergent-soluble and insoluble fractions were processed and immunoblotted with antibodies as previously described (30). The following primary antibodies were used: a rabbit polyclonal antibody against human PNLIP generated in our lab (1:5,000) (17), a rabbit polyclonal antibody against BiP (1:1,000; Cat.: 3183, Cell Signaling), a rabbit polyclonal antibody against GRP94 (1:1,000; Cat.: 2104, Cell Signaling), and a rat monoclonal antibody against α-tubulin (1:2,000; Cat.: sc-53029, Santa Cruz Biotechnology) used as an endogenous control. The secondary antibodies included an IRDye 680RD goat anti-rabbit IgG (1:10,000; Cat.: 926-68071) and an IRDye 800CW goat anti-rat IgG (1:10,000; Cat.: 926-32219) from LI-COR. Images of the immunoblots were captured with ChemiDoc MP Imaging System, and band densitometry analysis was performed using Image Lab (Bio-Rad).

### RNA extraction, RT-PCR, and qPCR

Total RNA was extracted from transfected cells using the RNeasy Plus Mini Kit (Cat.: 74134, Qiagen) and then reverse-transcribed into complementary DNA (cDNA) as previously described (34). The resulting cDNA served as the template for qPCR, which was performed using TaqMan Fast Advanced Master Mix (Cat.: 4444964) on a QuantStudio 3 System. The probes used for the genes of interest included *PNLIP* (Hs00609591_m1), *BiP* (Hs00607129_gH), *CHOP* (Hs00358796_g1), and *18S* (Hs03003631_g1), which served as an endogenous control. All probes were obtained from Thermo Fisher. The cycle threshold (Ct) values obtained were used to calculate the expression fold change relative to the endogenous control. The expression values for the *PNLIP* S290N group were then normalized to the mean values for the WT control group.

X-box binding protein-1 (*Xbp1*) mRNA splicing was assessed by semi-quantitative PCR as previously described (17). The amplified products were 441 bp for the unspliced form and 415 bp for the spliced form. PCR products were run on 2% agarose gels, and bands were visualized using ethidium bromide staining. The band density was quantified by densitometry. The extent of *Xbp1* mRNA splicing was calculated as the ratio of the density of the spliced *Xbp1* band to the sum of the densities of both the spliced and unspliced bands.

### Data analysis

Data analysis was performed using the GraphPad Prism 10.0.1 software package. One-way ANOVA mixed-effects analysis was conducted to perform Tukey's multiple comparisons, with *P* < 0.05 being considered statistically significant.

## Results

### Study participants

**Fig. 1:**
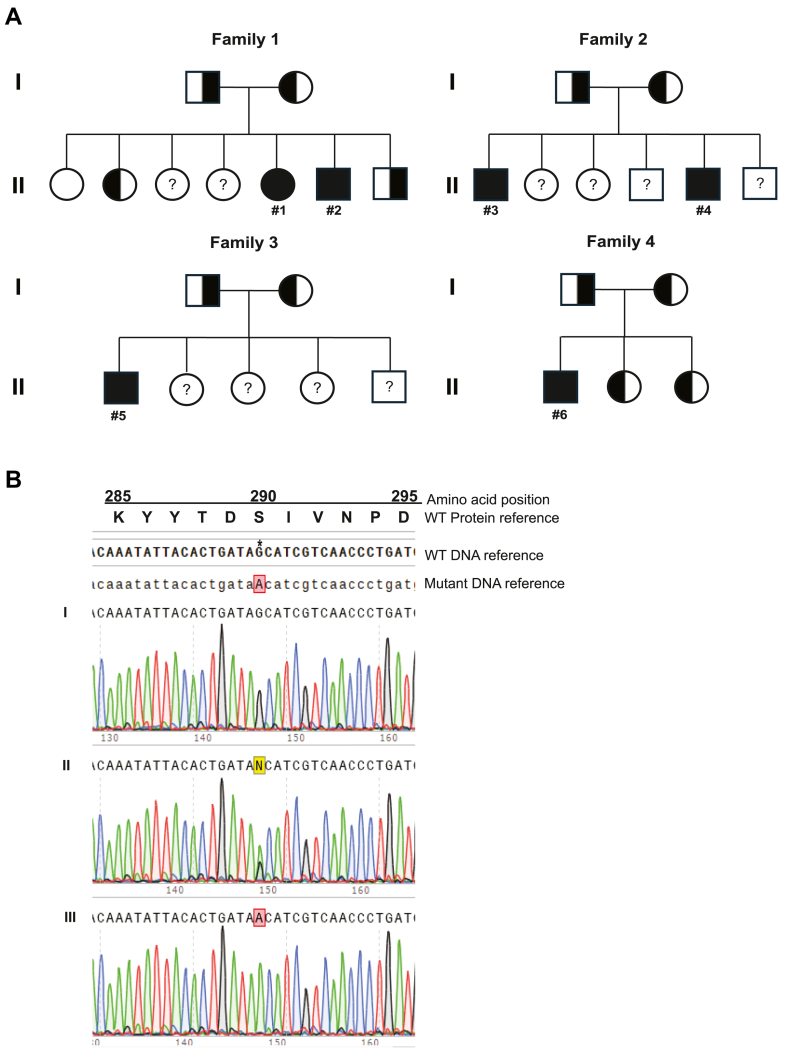
Identification of the novel *PNLIP* c.869G>A (p.S290N) variant by Sequencing. A: Pedigrees of affected Families with symbols representing family members. Squares represent males, and circles represent females. Open, filled, and half-open-half-filled symbols indicate individuals with the wild-type (WT) genotype, homozygous for the variant allele, and heterozygous for the variant allele, respectively. Open symbols with a question mark (?) indicate unaffected individuals who have not been genetically tested for the variant allele. Patients #1–6 are labeled. B: Representative sequencing chromatograms of Family 1. I: An unaffected sister with the WT genotype for the variant. II: An unaffected parent who is a heterozygous carrier of the variant. III: Patient #1 with the homozygous variant. Position c.869 (p.S290) is marked by the symbol ∗.

A total of six children from four Old Order Amish descent were evaluated for stool abnormalities. **Family 1**: Patient #1, a now 16-year-old female, presented at 6 years of age with complaints of diarrhea and foul-smelling, oily stools that were first noted at 10 months of age following the introduction of table foods (Fig. 1A and Table 1). Previous evaluations did not reveal etiology. Genetic tests for cystic fibrosis, cartilage hair hypoplasia, alpha-1-antitrypsin deficiency, and cholestatic diseases were negative. The patient was empirically started on Creon (AbbVie) as pancreatic enzyme replacement therapy (PERT) at 4 years of age, and her symptoms resolved. Patient #2, the 11-year-old brother of Patient #1, presented at 22 months of age with similar symptoms and medical history. The cholestasis multigene panel was negative. The brother's symptoms also resolved with the use of PERT. The parents and five siblings are asymptomatic.

**Table 1:** Laboratory test results and growth parameters of the patients

	Family 1		Family 2		Family 3	Family 4
Patient #	#1	#2	#3	#4	#5	#6
Sex	Female	Male	Male	Male	Male	Male
Age at presentation (month)	10	10	13	11	18	10–12
Age at genetic diagnosis (year)	12	8	4	2	6	17
Weight (percentile)	7	25	77	78	8.4	53
Height (percentile)	5	7	72	82	5.4	51
BMI (kg/m^2^)	18.3	17.8	17.1	16.7	15.09	21.58
Serum Lipase (15–52 U/L)	8	11	8	9	Not done	5
Vitamin A (26-72 mcg/dl)	26	21	21	30	Not done	62
Vitamin D (30–100 ng/ml)	23	31	42	41	Not done	20
Vitamin E γ-tocopherol (≥3.8 mg/L)	1.3	1.0	<1.0	<1.0	Not done	<1.0
Vitamin E α-tocopherol (mg/L)	4.7 (3.7–12.4)	3.1 (3.7–12.4)	3.3 (5.5–11.8)	<1.0 (2.9–16.6)	Not done	8.0 (2.9–16.6)
Fecal Elastase (>200 μg/g)	92	Not done	99	Not done	Not done	Not done
Pancreas ultrasound	Normal	Not done	Not done	Not done	Not done	Not done

**Family 2**: Patient #3, an 8-year-old male with Hemophilia B (Factor IX), presented at 3 years of age with oily, orange-colored stools, abdominal pain, and flatulence (Fig. 1A and Table 1). His symptoms started at 13 months of age. Patient #4, the 6-year-old brother of Patient #3, presented at 2 years of age with diarrhea and oily stools associated with the introduction of solid foods. The brothers' work-up included a six-gene hereditary pancreatitis panel and a cholestasis multigene panel. Both were unrevealing. The parents and four siblings are asymptomatic. Family 2 reported a paternal family history of distant relatives with similar gastrointestinal symptoms, including the father's cousin's daughter and the paternal grandfather's cousin's daughter. The brothers' symptoms resolved with PERT.

**Family 3**: Patient #5, a 6-year-old male, presented with loose, oily, foul-smelling stools and oily discharges (Fig. 1A and Table 1). His symptoms began at around 18 months old after toilet training, accompanied by stomach rumbling and flatulence. He has four unaffected siblings. The parents and their siblings are generally healthy. The parents' nieces and nephews are healthy, except for a maternal niece and a paternal nephew who have heart disease. Both sets of grandparents are living and healthy. The paternal grandfather has family members with hypertrophic cardiomyopathy. The parents were originally from Indiana, and the family resided in Wisconsin. The family had corresponded with either Family 1 or 2 regarding similar concerns over oily stools. The patient was given PERT and a high ADEK multivitamin supplement, resolving symptoms.

**Family 4**: Patient #6 is a 16-year-old male who presented with stool abnormalities (Fig. 1A and Table 1). His symptoms began around 10–12 months of age when table foods were introduced. The patient's parents are consanguineous, being fourth cousins, and he has two unaffected sisters. The parents and his siblings are generally healthy. Initially, the patient was diagnosed with cystic fibrosis, though it is unclear whether this diagnosis was based on sweat chloride testing, genetic testing, both, or neither. This diagnosis was later revised to a pancreatic disorder. Additionally, the patient's paternal cousin's two children, identified as patient #3 and patient #4 from Family 2, were diagnosed with pancreatic lipase deficiency earlier. The patient was treated with PERT, which improved his symptoms.

Recent growth percentiles and laboratory values are presented in Table 1. Patients #3, #4, and #6 were above the 50th percentile for weight and height, whereas Patients #1, #2, and #5 were below the 50th percentile for both. All patients had normal BMI values for their respective ages, except Patient #5 who was underweight. All patients except Patient #5, who was not tested, had lower-than-normal serum lipase levels and were deficient in Vitamin E. The Vitamin A levels for Patients #1-4 were at or below the lower range of normal, and normal for Patient # 6, with Patient #5 not being tested. Additionally, Patients #1 and 6 had mild Vitamin D deficiency, and Patients #2-4 had normal Vitamin D levels, with Patient #5 not being tested. Fecal elastase values for Patients #1 and #3 were in the exocrine pancreatic insufficiency (EPI) range, and the other four patients were not tested. Only Patient #1 had an abdominal ultrasound, which revealed no pancreatic abnormalities.

### Variant identification

Whole exome sequencing was employed to identify the cause of the symptoms after multiple targeted gene tests were unrevealing. Exome sequencing uncovered a novel homozygous single-base substitution in exon 9 of the *PNLIP* gene (NG_023311.1: g.15142G>A; NM_000936.4: c.869G>A) in both affected probands from Family 1. The substitution introduces a missense variant at amino acid position 290 (NP_000927.1: p.Ser290Asn or p. S290N). Targeted Sanger sequencing confirmed the affected siblings were homozygous for the p.S290N variant allele and revealed that their parents and brother were heterozygous for the variant allele (Fig. 1A and B). Among their four sisters, one was heterozygous for the variant allele, one was homozygous for the WT reference allele, and the other two were not tested. The association of the *PNLIP* p.S290N variant with CPLD was further supported by the identification of the homozygous p.S290N variant in Patients #3 and 4 from Family 2, Patient #5 from Family 3, and Patient # 6 from Family 4. For Families 2 and 3, the unaffected parents were all heterozygous for the variant, and the other unaffected family members were not tested for the variant allele. For Family 4, both unaffected parents and two siblings were heterozygous for the variant allele. The clear segregation of the homozygous *PNLIP* p.S290N variant with the clinical symptoms provides convincing evidence that the variant causes autosomal recessive CPLD.

These four families represent the first reported cases of a *PNLIP* pathogenic variant associated with CPLD in the Amish population. According to the Genome Aggregation Database (gnomAD), the *PNLIP* p.S290N variant (dbSNP, rs1332713285) has an allele frequency of 0.0000223 (36 out of 1,613,882 alleles as of 7/24/2025). The highest allele frequency is observed in European (non-Finnish) populations at 0.00002882. This variant has not yet been documented in other ethnicities, including African/African American, Admixed American, Ashkenazi Jewish, East and South Asian, and Middle Eastern, indicating a strong ethnic specificity likely due to founder effect and subsequent genetic drift.

### Computational modeling of variant impact

**Fig. 2:**
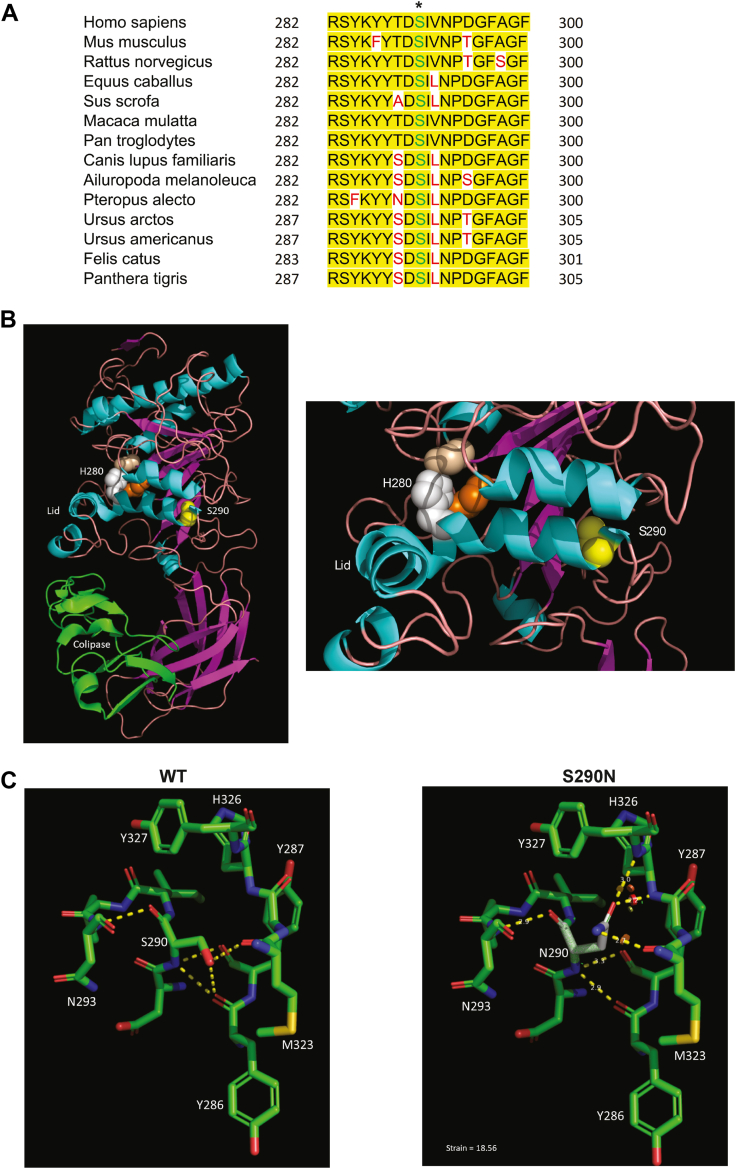
Protein sequence analysis of the novel p.S290N variant PNLIP. A: Multiple-sequence alignment analysis of the region around p.S290 of PNLIP proteins across species. Position c.869 (p.S290) is marked by the symbol ∗. B: Modeling of PNLIP protein structure complexed with colipase. C: Comparison of WT and the p.S290N variant PNLIP protein structures. WT, wild type.

Sequence analysis of the PNLIP protein products shows that the Ser290 residue is highly conserved across species, indicating its potential importance in maintaining the proper structural conformation and function of PNLIP (Fig. 2A). We utilized multiple structure prediction tools to predict the effect of the p.S290N substitution on the structure of PNLIP. PredictSNP1 classified the variant as deleterious with an expected accuracy of 87%. SAAPdap/SAAPpred used three crystal structures of PNLIP (1lpa, 1lpb, and 1n8s) to predict that p.S290N is pathogenic with 0.40 confidence. AlphaMissense predicted that p.S290N was likely pathogenic with a pathogenicity score of 0.931. Altogether, these algorithms indicate that p.S290N is pathogenic.

To determine the potential mechanism of pathogenicity, we modeled p.S290N in one structure (1lpb) (35). This structure shows PNLIP is complexed with colipase (Fig. 2B). The lid domain, which moves to cover or expose the p.S169-H280-A193 catalytic triad, is in the open position (35). p.S290 is in the same alpha helix as p.H280. The helix also contributes to the lid domain. The α-carbon of p.S290 is 18.9 Å from the α-carbon of p.S169. Modelling p.N290 into PNLIP shows an alteration in hydrogen bonds and the presence of steric hindrance (Fig. 2C). These changes likely affect the local structure and alter the conformation of p.H280 and the lid, leading to the damaging effect of p.S290N on the stability of PNLIP.

### Variant impact on function and protein folding

**Fig. 3:**
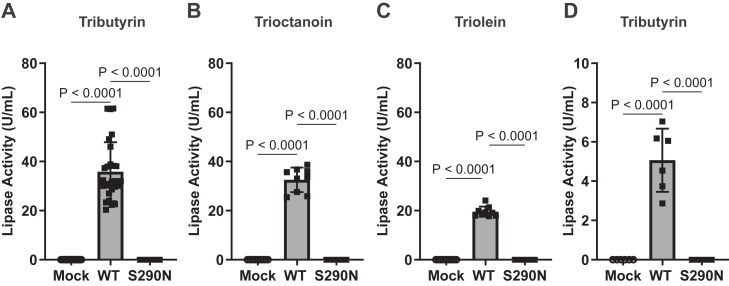
The p.S290N variant led to functional loss of PNLIP in vitro. HEK 293T cells were transfected to express PNLIP p.S290N and WT. Samples were harvested 68 h post-transfection and measured for lipase activity as described in the Methods. Lipase activity of conditioned media against various substrates (A–C). A: Tributyrin. B: Trioctanoin. C: Triolein. D: Lipase activity of detergent-soluble fraction of cell lysates against tributyrin. Mock, empty vector; WT, wild type. The values in quantification graphs are expressed as the mean ± S.D. of 6 or more independent experiments, and One-Way ANOVA Mixed-effects analysis by Tukey's multiple comparisons were performed.

We next assessed the impact of the p.S290N variant on the functional properties of PNLIP in cell culture. HEK 293T cells were chosen for their ease of handling and manipulation. More importantly, prior studies from our group and others have characterized genetic risk variants associated with pancreatitis in genes encoding digestive enzymes using both HEK 293T cells and AR42J cells, a rat pancreatic acinar cell line. The results obtained from these two in vitro systems were highly similar, suggesting that the findings gained from HEK 293T cell system have close physiological relevance when assessing the impact of variants on digestive enzymes (20, 31, 36, 37). The cells were transfected with empty plasmid or a plasmid expressing either PNLIP WT or PNLIP p.S290N. First, we measured the lipase activity in the conditioned media from transfected cells (Fig. 3A–C). No lipase activity was detected in the mock group against tributyrin, trioctanoin, or triolein, while the PNLIP WT group exhibited robust lipase activity against all three substrates. Notably, the p.S290N variant completely abolished lipase activity in the media. We then measured the lipase activity in cell lysates using tributyrin (Fig. 3D). No lipase activity was detected in the mock group. Strong lipase activity was observed in the PNLIP WT group. No lipase activity was detected in the PNLIP p.S290N samples. These data demonstrate that the p.S290N variant leads to loss of PNLIP lipase function.

**Fig. 4:**
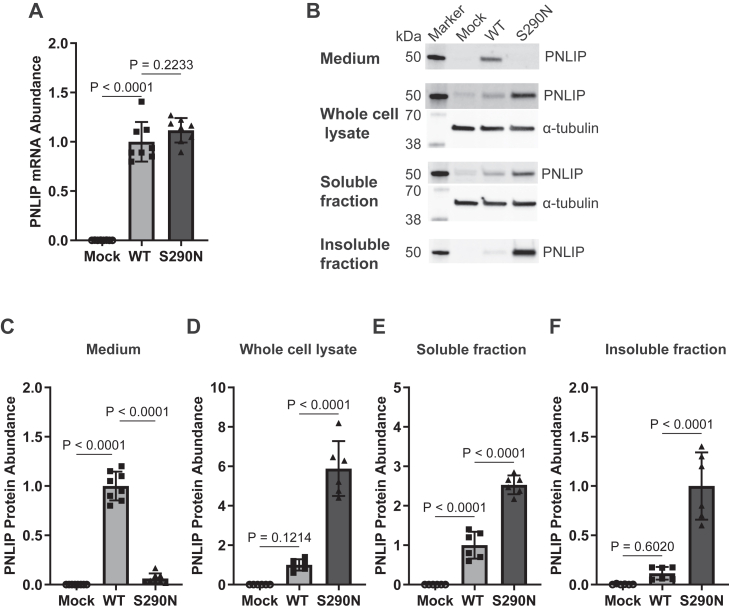
The p.S290N variant led to intracellular retention and secretion defect of PNLIP in vitro. HEK 293T cells were transfected to express PNLIP p.S290N and WT. Samples were harvested 68 h post-transfection for qPCR and immunoblotting for PNLIP as described in the Methods. (A) Relative mRNA abundance of PNLIP by qPCR. (B) Representative immunoblot images for PNLIP. α-tubulin served as a control. The relative PNLIP protein abundance in different partitions were quantified relative to WT, with the exception for insoluble fraction where the values were expressed as relative to S290N. (C) Medium. (D) Whole cell lysate. (E) Detergent-soluble fraction. (F) Detergent-insoluble fraction. Mock, empty vector; WT, wild type. The values in quantification graphs are expressed as the mean ± S.D. of 6 or more independent experiments, and One-Way ANOVA Mixed-effects analysis by Tukey's multiple comparisons were performed.

Next, we confirmed by qPCR that while no detectable PNLIP expression was present in the mock group, PNLIP expression at the mRNA level was markedly increased in the PNLIP WT and p.S290N groups and was comparable between these two groups (Fig. 4A). These data support that the loss of lipase function in the variant group shown in Figure 3 is due to the impact of the variant itself, rather than a lack of expression. Furthermore, the similar expression levels of PNLIP between the WT and variant groups enable proper comparison for the following studies. We then determined whether the p.S290N variant impacts PNLIP protein folding by assessing its partitioning in the medium, whole cell lysate, detergent-soluble fraction, and detergent-insoluble fraction. Immunoblot analysis demonstrated the clear presence of PNLIP in the conditioned medium from the PNLIP WT group, but not in the mock or p.S290N group (Fig. 4B and C). Conversely, significantly higher levels of PNLIP protein were present in the whole cell lysates from the p.S290N group compared to the mock and WT groups (Fig. 4B and D). When the whole cell lysate was separated into detergent-soluble and insoluble fractions, increased amounts of PNLIP p.S290N were present in both the detergent-soluble and -insoluble fractions than were present in the samples from the mock and WT transfected cells (Fig. 4B, E, and F). The lack of secretion and intracellular retention of PNLIP p.S290N suggests that the variant protein misfolds inside the cells.

Due to the very low secretion of the variant PNLIP in the medium, it is not feasible for us to purify the variant lipase to conduct biochemical function characterization and biophysical studies of PNLIP p.S290N compared to PNLIP WT. Nevertheless, given the higher amount of PNLIP protein in the detergent-soluble fraction of the transfected cells in the variant group compared to the PNLIP WT group (Fig. 4B and E), the absence of lipase activity in the detergent-soluble fraction in the PNLIP p.S290N group (Fig. 3D) implies that the variant causes a catalytic defect in the lipase. Additionally, the majority of PNLIP p.S290N protein is in the detergent-insoluble fraction of transfected cells compared with nearly no PNLIP WT protein in this fraction, supporting the conclusion that the missense mutation affects the folding of the variant lipase (Fig. 4B and F).

### Variant impact on cellular response

PNLIP is one of the most abundant digestive enzymes produced by pancreatic acinar cells (10). An increased burden of misfolded protein caused by the expression of PNLIP p.S290N may result in increased ER stress and an unmitigated UPR leading to cell death. Therefore, we investigated whether the p.S290N variant leads to increased ER stress and activation of the UPR.

**Fig. 5:**
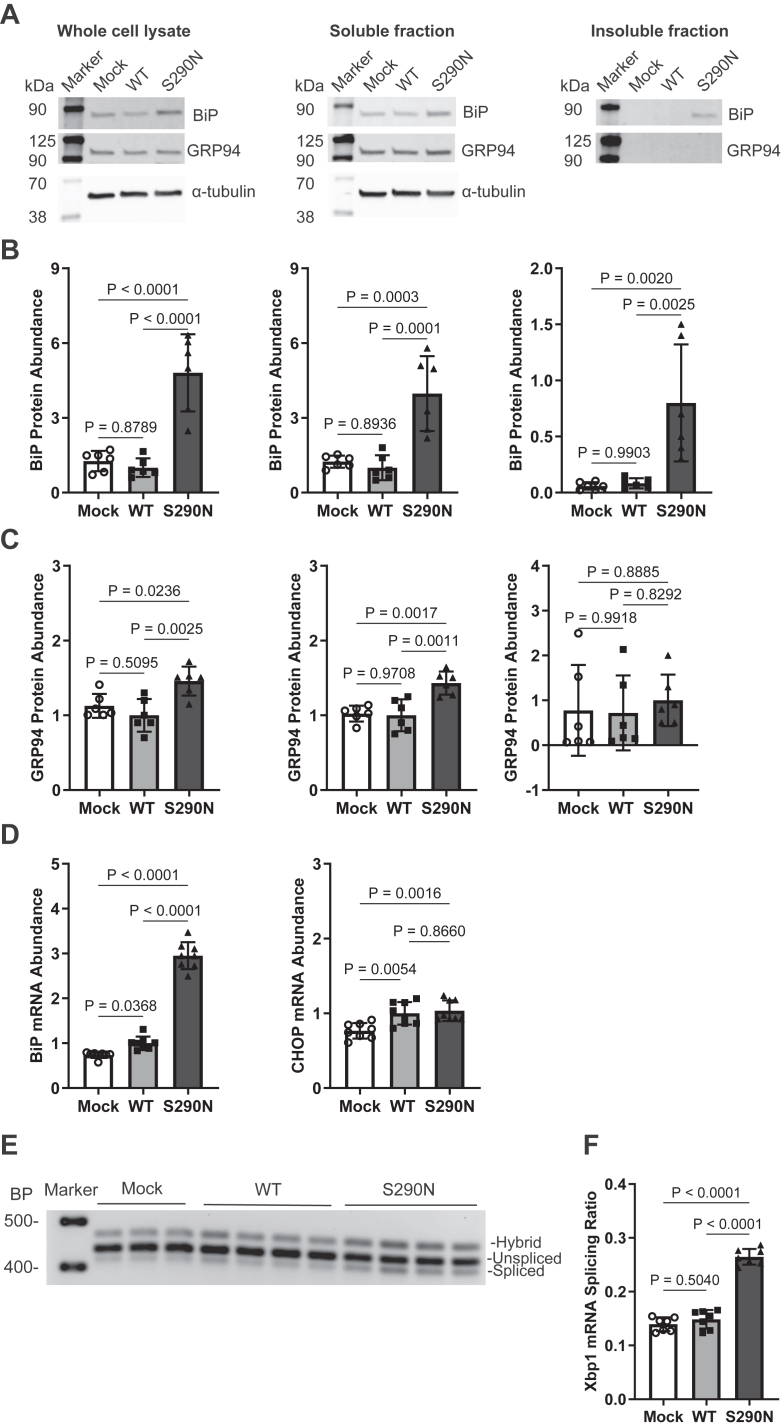
Expression of the PNLIP p.S290N variant induced increased ER stress and the UPR in vitro. HEK 293T cells were transfected to express PNLIP p.S290N and WT. Samples were harvested 68 h post-transfection for immunoblotting and qPCR analysis for ER stress and the UPR markers as described in the Methods. A: Representative immunoblot images for various markers. α-tubulin served as a control. The relative protein abundance for each marker in different cell lysate fractions presented in (A) was quantified relative to WT, with the exception for the insoluble fraction where the values were expressed as relative to S290N. B: Relative BiP protein abundance. C: Relative GRP94 protein abundance. D: Relative mRNA abundance of BiP and CHOP by qPCR. E: A representative image of semi-quantitative PCR analysis of *Xbp1* mRNA splicing. F: Quantification of *Xbp1* mRNA splicing presented in (E). Mock, empty vector; WT, wild type. The values in quantification graphs are expressed as the mean ± S.D. of 6 or more independent experiments, and One-Way ANOVA Mixed-effects analysis by Tukey's multiple comparisons were performed.

We first measured the protein abundance of BiP and GRP94, two major biomarkers for ER stress, in transfected cells using immunoblot analysis in the whole cell lysate and in the detergent-soluble and -insoluble fractions (Fig. 5A–C). While the expression of PNLIP WT protein did not cause a significant increase in BiP and GRP94 in any fraction compared to the mock control group, expression of PNLIP p.S290N caused a significant increase in both BiP and GRP94 relative to the WT control in the whole cell and detergent-soluble fractions. Furthermore, there was a significant increase in BiP, but not GRP94, in the insoluble fraction, suggesting an active and specific role of BiP in handling the misfolded PNLIP variant. The upregulation of BiP in the variant group was further supported by a significant increase in *BiP* mRNA abundance as assessed by qPCR (Fig. 5D). There was no difference in CHOP mRNA abundance between the WT and PNLIP variant groups. However, semi-quantitative PCR analysis of *Xbp1* mRNA splicing, a classic marker for the UPR, revealed significantly increased *Xbp1* mRNA splicing in the variant group compared to the WT control group (Fig. 5E and F). Collectively, these data suggest that the *PNLIP* p.S290N variant has the potential to elicit increased ER stress and activation of the UPR to cause proteotoxicity.

### Function-defective PNLIP variants

Over the past decade, our group has characterized various newly identified PNLIP variants (20, 21, 32, 34, 38). A list of these characterized PNLIP variants is shown in Table 2. Although most of the variants exhibit normal or nearly normal functions when assayed, a fraction of the variants demonstrates defective function. This dysfunction can be due to defective secretion, catalytic inactivation, or both. The potentially pathogenic *PNLIP* variants, which impact lipase activity involved in fat digestion, include p.H92N, p.W102∗, p.A174P, p.R188C, p.C198Y, p.T221M, p.G233E, p.C254R, p.D264Y, p.S290N, and p.W419∗.

**Table 2:** Summary of previously characterized variants in the *PNLIP* gene

Exon	Nucleotide Change	AA Change	SNP	Zygosity	Residual Secretion/Specific Activity^a^	Cited
3	c.124C>T	p.H42Y	NA	Het	∼95%/∼75%	(32)
3	c.160C>T	p.R54C	rs142749694	Het	∼90%/∼90%	(32)
3	c.172T>C	p.Y58H	rs76415321	Het	∼95%/∼90%	(32)
3	c.175A>G	p.T59A	rs147154871	Het	∼95%/∼90%	(32)
4	c.214G>T	p.D72Y	rs200008569	Het	∼95%/∼90	(32)
4	**c.274C>A**	**p.H92N**	**rs368162708**	**Het**	**∼95%/∼10%**	(32)
4	**c.305G>A**	**p.W102**^a^	**NA**	**Het**	**∼0%/∼0%**	(8)
5	c.427G>T	p.A143S	rs376332523	Het	∼95%/∼95%	(32)
6	c.487G>A	p.V163M	rs200040515	Het	∼95%/∼95%	(32)
6	**c.520G>C**	**p.A174P**	**rs201757600**	**Het**	**∼5%/ND**	(32, 41)
6	c.557T>C	p.I186T	rs78536862	Het/Hom(2)	∼95%/∼100%	(32)
6	**c.562C>T**	**p.R188C**	**rs199957067**	**Het**	**∼0%/∼0%**	(8)
7	**c.593G>A**	**p.C198Y**	**NA**	**Het**	**∼90%/∼10%**	(32)
7	**c.661C>T**	**p.T221M**	**rs746000327**	**Het/Hom(2)**	**∼0%/∼0%**	(7, 20, 34)
8	**c.698G>A**	**p.G233E**	**rs1373452993**	**Het**	**∼10%/ND**	(32, 41, 42)
8	c.733C>G	p.P245A	NA	Het	>75%/∼95%	(32)
8	**c.760T>C**	**p.C254R**	**rs750709623**	**Het**	**∼3%/ND**	(32, 41)
8	**c.790G>T**	**p.D264Y**	**rs755039876**	**Het**	**∼75%/∼5%**	(32)
8	c.794T>G	p.I265R	rs377358755	Het	∼90%/∼95%	(32)
8	**c.869G>A**	**p.S290N**	**rs1332713285**	**Het/Hom(6)**	**∼0%/∼0%**	
9	c.900C>A	p.F300L	rs890551695	Het	∼85%/∼70%	(32)
9	c.911C>T	p.S304F	NA	Het	∼75%/∼90%	(32)
9	c.919G>A	p.V307I	rs773774916	Het	∼80%/∼50%	(32)
10	c.942C>G	p.F314L	NA	Het	∼90%/∼85%	(32)
11	c.1102G>A	p.V368I	rs202075908	Het	∼95%/∼90%	(32)
12	c.1241T>A	p.M414K	rs111319548	Het	∼90%/∼70%	(32)
12	**c.1256G>A**	**p.W419**^a^	**rs765879360**	**Het/Hom(1)**	**∼0%/∼0%**	(9)
13	**c.1360G>T**	**p.V454F**	**rs148560679**	**Het**	**∼15%/∼50%**	(32, 41)

The variant in bold indicates that it is likely pathogentic in terms of lipase function.

AA, Amino acid; Het, Heterozygous; Hom, Homozygous, and the number in the parentheses indicates the count of homozygous carriers found; NA, Not available; ND, Not determined; SNP, Single nucleotide polymorphism.

a The residual secretion/specific activity of the PNLIP variant protein relative to that of PNLIP WT. The specific activity was assessed against triolein, the most common physiological substrate for PNLIP.

## Discussion

This study presents multiple cases of a novel homozygous *PNLIP* pathogenic variant associated with CPLD in the Old Order Amish population. All reported CPLD cases, including the current ones, support the autosomal recessive inheritance pattern of CPLD associated with *PNLIP* pathogenic variants. Affected individuals with abnormal stools typically have either homozygous or compound heterozygous variants, while heterozygous carriers often have normal stool fat (7, 8, 9). We provide multiple forms of evidence for the role of *PNLIP* p.S290N in causing the patients' symptoms. First, the homozygous missense variant segregates with the clinical phenotype in four families. Second, the findings that Ser290 is highly conserved across PNLIPs in various species and that computational modeling and analysis predict that the p.S290N substitution destabilizes the protein conformation underscore its importance in maintaining protein structure and function. Third, functional characterization confirms that the variant lipase misfolds intracellularly, resulting in defective secretion and loss of lipase activity. Lastly, the misfolded variant lipase accumulates inside the cells, increasing ER stress and activating the UPR, which could be proteotoxic and increase risk for chronic pancreatitis. Notably, all known variants associated with CPLD (p.R188C, p.T221M, p.S290N, and p.W419∗) except p.W102∗, which undergoes nonsense-mediated decay, exhibit protein misfolding and secretion defects.

These are the first reported cases of CPLD associated with homozygous *PNLIP* pathogenic variants found in non-consanguineous families (Families 1–3, but not Family 4) and the first cases of CPLD involving multiple Amish families carrying the *PNLIP* p.S290N variant. Of note, Family 2 has an extended paternal history of similar symptoms of abnormal stool and paternally related to Family 4. Family 1 or 3 are not closely related to other families, at least as second cousins. These findings suggest that the *PNLIP* p.S290N variant may be prevalent in this population, considering the known founder effect of inherited diseases among the Amish communities. Allele frequency analysis of the gnomAD suggests that the *PNLIP* p.S290N variant has strong European (non-Finnish) ethnicity-specificity, which further supports the probable founder origin of this variant in the Amish population. Given the unique cultural and healthcare practices within Amish communities, this report offers critical guidance for the identification and care of CPLD patients associated with the homozygous *PNLIP* p.S290N variant. Importantly, a rapid and inexpensive targeted sequencing method has been established at the Clinic for Special Children to test for this variant. This targeted genetic test can be used to test Amish individuals who exhibit stool abnormalities, such as greasy stools. Or in Amish families at high risk for having CPLD, infants can be screened for this variant. If they are confirmed to be homozygous for the *PNLIP* p.S290N variant, PERT can be prescribed to alleviate the symptoms. Additionally, individuals, particularly older adults, who are confirmed to be homozygous for the *PNLIP* p.S290N variant should be closely monitored for potential signs of chronic pancreatitis and diabetes.

Consistent with previously reported cases of CPLD, all six patients demonstrated nearly normal growth, development, and body mass index despite experiencing steatorrhea. Steatorrhea was first noticed in affected individuals around 1–2 years of age with the introduction of solid foods. This age of onset aligns with other reported CPLD cases, where symptoms typically begin in late infancy. There are two possible reasons for the absence of steatorrhea during early infancy. First, CEL, also known as bile salt-stimulated or activated lipase, which is present in mother's milk, may compensate for PNLIP deficiency during early infancy (14, 39). Second, our work has shown that PNLIP may be temporally absent in the pancreas during early life, while PNLIPRP2 may play an important role in fat digestion during this period, along with CEL(15–19). Therefore, the S290N variant does not impact fat digestion when CEL and PNLIPRP2 may compensate for the lack of contribution from PNLIP. When used, PERT significantly improved fat digestion and alleviated associated clinical symptoms in all reported cases.

There are two major limitations in our study. First, the in vitro characterization of the variant was conducted using HEK 293T cells, a non-pancreatic acinar cell line. Previous investigations into variants in genes encoding digestive enzymes, including our studies on the PNLIP p.T221M and CEL-MODY8 variants, as well as CPA1 and CTRC variants studied by others, showed similar results in terms of protein misfolding, secretion defects, and resultant proteotoxicity when both HEK 293T cells and pancreatic acinar cell lines, such as AR42J cells, were used (20, 31, 36, 37). These prior studies suggest that HEK 293T cells are suitable for characterizing misfolding variants in digestive enzymes. However, employing pancreatic acinar cells may yield more physiologically relevant insights. These cell lines include AR42J cells, 266-6 (a mouse pancreatic acinar cell line), or primary pancreatic acinar cells from mice or humans. A caveat is that pancreatic acinar cells are notoriously difficult to transfect using common reagents. The most common method for expressing foreign proteins in these cells is recombinant adenovirus transduction, which is time-consuming and costly. Another minor issue with using pancreatic acinar cells is the endogenous expression of digestive enzymes such as PNLIP, which may complicate analysis and data interpretation. Certainly, in vivo studies of the variant would be more physiologically relevant, as they eliminate issues involving expression levels and inherent problems in maintaining pancreatic acinar cell architecture and characteristics in in vitro culture system. Considering the 290Ser residue is highly conserved across species, including mice, creating a knock-in mouse model of the variant would help provide the most straightforward and physiological relevant findings in the future. The knock-in mouse model would allow us to fully characterize the impact of this variant under physiological conditions, including protein misfolding, secretion defects, cellular localization of the variant protein, ER stress, and the UPR response. Notably, the data from the present study suggest that the variant PNLIP is retained intracellularly, yet we did not pinpoint the precise cellular localization of the variant lipase inside the cell, either within the ER or other subcellular compartments.

The second limitation is the clinical implication of the potential proteotoxicity of the p.S290N variant, which leads to PNLIP misfolding, eliciting ER stress and increased UPR in vitro. As discussed above, the knock-in mouse model of this variant would offer more physiologically relevant insight regarding whether the variant could be proteotoxic. Interestingly, both the patients homozygous for the *PNLIP* p.T221M or p.S290N variant have abnormal fecal elastase levels consistent with EPI (7). Notably, in the two affected brothers who are homozygous for the p.T221M variant, the pancreolauryl test was abnormal, indicating impaired exocrine pancreatic function beyond isolated pancreatic lipase deficiency (7). In contrast, a patient with the homozygous p.W419∗ variant has pancreatic lipase deficiency but normal levels of fecal elastase (9). In addition, the p.W419∗ patients have no pancreatic abnormalities on imaging. No imaging had been conducted on the affected brothers with *PNLIP* p.T221M, and one of the p.S290N patients showed a normal pancreas on abdominal ultrasound. Although our *Pnlip* p.T221M mice develop chronic pancreatitis supporting the hypothesis that *PNLIP* p.T221 M can increase the risk for chronic pancreatitis through a protein misfolding and ER stress-related mechanism, differences between mouse and human are widely recognized (34). It is unclear whether normal pancreatic imaging in one of the affected patients suggests that the disease is not yet advanced enough to be detected by imaging. Still, the findings of low levels of fecal elastase and the potential for *PNLIP* variants to cause chronic pancreatitis suggest clinicians caring for these patients should be aware of the possibility that these patients may develop chronic pancreatitis, especially as they age. Interestingly, abdominal pain, a common symptom in chronic pancreatitis, is not prominent in patients with misfolding *PNLIP* variants (7). Of note, patients with the *CEL*-MODY8 variant develop chronic pancreatitis, and abdominal pain is also uncommon in these patients (40).

In conclusion, the findings of our study expand the clinical literature on CPLD associated with *PNLIP* variants and offer guidance for improved diagnosis and care of CPLD patients with this novel pathogenic variant, particularly in the Amish communities. Our data indicate that the *PNLIP* p.S290N variant results in CPLD through a loss-of-function phenotype, characterized by abolished catalytic activity and defective secretion due to protein misfolding. Further investigations are warranted to determine whether the misfolded variant induces proteotoxicity, potentially increasing the risk of pancreatic injury and CP.

## Data availability

The data that support the findings of this study are available from the corresponding author (XKX) upon reasonable request.

## Conflict of interest

The authors declare that they do not have any conflicts of interest with the content of this article.
